# Supplementary material for: An evolutionarily conserved stop codon enrichment at the 5′ ends of mammalian piRNAs
Source: Nat Commun. 2022 Apr 19;13:2118. doi: 10.1038/s41467-022-29787-3 (PMC9018710; doi:10.1038/s41467-022-29787-3)
Supplement: Supplementary file 2 — Description of Additional Supplementary Files [file 41467_2022_29787_MOESM2_ESM.pdf]

Description of Additional Supplementary Files

File Name: Supplementary Data 1
Description: List of testis and ovary libraries per species in the piRNA cluster database.
