# Supplementary material for: An evolutionarily conserved stop codon enrichment at the 5′ ends of mammalian piRNAs
Source: Nat Commun. 2022 Apr 19;13:2118. doi: 10.1038/s41467-022-29787-3 (PMC9018710; doi:10.1038/s41467-022-29787-3)
Supplement: Supplementary file 4 — Reporting Summary [file 41467_2022_29787_MOESM4_ESM.pdf]

nature portfolio

Corresponding author(s): Susanne Bornelöv; Gregory J Hannon

Last updated by author(s): Feb 4, 2022

Reporting Summary

Nature Portfolio wishes to improve the reproducibility of the work that we publish. This form provides structure for consistency and transparency in reporting. For further information on Nature Portfolio policies, see our Editorial Policies and the Editorial Policy Checklist.

Statistics

For all statistical analyses, confirm that the following items are present in the figure legend, table legend, main text, or Methods section.

n/a	Confirmed	
☐	☒	The exact sample size (*n*) for each experimental group/condition, given as a discrete number and unit of measurement
☐	☒	A statement on whether measurements were taken from distinct samples or whether the same sample was measured repeatedly
☐	☒	The statistical test(s) used AND whether they are one- or two-sided<br>*Only common tests should be described solely by name; describe more complex techniques in the Methods section.*
☒	☐	A description of all covariates tested
☒	☐	A description of any assumptions or corrections, such as tests of normality and adjustment for multiple comparisons
☐	☒	A full description of the statistical parameters including central tendency (e.g. means) or other basic estimates (e.g. regression coefficient) AND variation (e.g. standard deviation) or associated estimates of uncertainty (e.g. confidence intervals)
☐	☒	For null hypothesis testing, the test statistic (e.g. *F*, *t*, *r*) with confidence intervals, effect sizes, degrees of freedom and *P* value noted<br>*Give P values as exact values whenever suitable.*
☒	☐	For Bayesian analysis, information on the choice of priors and Markov chain Monte Carlo settings
☒	☐	For hierarchical and complex designs, identification of the appropriate level for tests and full reporting of outcomes
☐	☒	Estimates of effect sizes (e.g. Cohen's *d*, Pearson's *r*), indicating how they were calculated

Our web collection on statistics for biologists contains articles on many of the points above.

Software and code

Policy information about availability of computer code

Data collection	Data were obtained from public repositories or databases.
Data analysis	High-throughput data was processed using Trim Galore! (v0.6.4), Bowtie (v1.2.3), TopHat2 (v2.1.1), bedtools (v2.26.0), BEDOPS (v2.4.37), samtools (v1.6). The custom scripts used to process the piRNA cluster database are available at GitHub: https://github.com/susbo/5prime_stop_paper. The R programming language (v3.6.3) was used for all statistical analyses and to prepare graphs.

For manuscripts utilizing custom algorithms or software that are central to the research but not yet described in published literature, software must be made available to editors and reviewers. We strongly encourage code deposition in a community repository (e.g. GitHub). See the Nature Portfolio guidelines for submitting code & software for further information.

Data

Policy information about availability of data

All manuscripts must include a data availability statement. This statement should provide the following information, where applicable:

- Accession codes, unique identifiers, or web links for publicly available datasets
- A description of any restrictions on data availability
- For clinical datasets or third party data, please ensure that the statement adheres to our policy

For mouse we used the mm10 genome assembly, dbSNP build 150 and Gencode vM24 gene models. Conservation scores, tRNA, and rRNA annotations were downloaded from the UCSC genome browser (downloaded 2020-02-10). For all other species we used the genome assembly and annotations listed in the piRNA cluster database. Mouse data used in this study are available in the GEO database under accession codes GSE20327 [https://www.ncbi.nlm.nih.gov/geo/query/acc.cgi?acc=GSE20327], GSE65786 [https://www.ncbi.nlm.nih.gov/geo/query/acc.cgi?acc=GSE65786], GSE107832 [https://www.ncbi.nlm.nih.gov/geo/query/acc.cgi?acc=GSE107832], GSE150350 [https://www.ncbi.nlm.nih.gov/geo/query/acc.cgi?acc=GSE150350], or the SRA database under accession codes PRJNA421205

[https://www.ncbi.nlm.nih.gov/sra/?term=PRJNA421205], PRJNA194540 [https://www.ncbi.nlm.nih.gov/sra/?term=PRJNA194540], PRJNA111011 [https://www.ncbi.nlm.nih.gov/sra/?term=PRJNA111011], PRJDB4628 [https://www.ncbi.nlm.nih.gov/sra/?term=PRJDB4628]. Data from other species used in this study are available from the piRNA cluster database (https://www.smallrnagroup.uni-mainz.de/piRNAclusterDB). Source data are provided with this paper.

Field-specific reporting

Please select the one below that is the best fit for your research. If you are not sure, read the appropriate sections before making your selection.

☒ Life sciences ☐ Behavioural & social sciences ☐ Ecological, evolutionary & environmental sciences

For a reference copy of the document with all sections, see nature.com/documents/nr-reporting-summary-flat.pdf

Life sciences study design

All studies must disclose on these points even when the disclosure is negative.

Sample size	We used all available replicates from the respective data sources.
Data exclusions	For the piRNA cluster database analysis, five samples were excluded for technical reasons (wrong species or failed pre-processing). Moreover, libraries with extremely few reads were excluded in some analyses. All data exclusions are stated in the manuscript.
Replication	The analyses were performed using both individual and pooled/averaged samples. The number of replicates used is stated in each analysis in manuscript and individual replicates are shown whenever feasible. For mouse data, the number of replicates available per experimental condition was typically between 1 and 4 (as shown in Fig. 1 Fig. 2, and Supplementary Fig. 2). For the conservation analysis, we relied on the number of libraries available per species and tissue combination (between 1 and 10, as listed in Supplementary Data 1). The results were reproducible across individual replicates unless otherwise stated.
Randomization	We have not performed any allocation into experimental groups, and randomization is therefore not applicable.
Blinding	All sequencing libraries were processed by the same pipeline that does not rely on any sample annotations.

Reporting for specific materials, systems and methods

We require information from authors about some types of materials, experimental systems and methods used in many studies. Here, indicate whether each material, system or method listed is relevant to your study. If you are not sure if a list item applies to your research, read the appropriate section before selecting a response.

Materials & experimental systems

n/a	Involved in the study	
☒	☐	Antibodies
☒	☐	Eukaryotic cell lines
☒	☐	Palaeontology and archaeology
☒	☐	Animals and other organisms
☒	☐	Human research participants
☒	☐	Clinical data
☒	☐	Dual use research of concern

Methods

n/a	Involved in the study	
☒	☐	ChIP-seq
☒	☐	Flow cytometry
☒	☐	MRI-based neuroimaging
